# Potential increase in Crimean-Congo hemorrhagic fever incidence in Iraq Post Eid-al-Adha, 2023

**DOI:** 10.1016/j.nmni.2023.101175

**Published:** 2023-09-07

**Authors:** Safin Hussein, Karzan Qurbani, Sirwan Khalid Ahmed

**Affiliations:** Department of Biology, College of Science, University of Raparin, Rania, Sulaymaniyah, 46012, Iraq; Department of Adult Nursing, College Nursing, University of Raparin, Rania, Sulaymaniyah, Kurdistan Region, 46012, Iraq; Ministry of Health, General Directorate of Health-Raparin, Rania, Sulaymaniyah, Kurdistan Region, 46012, Iraq

**Keywords:** Animals, CCHF, Eid-al-Adha, Iraq, Transmission

Dear Editor,

Crimean-Congo Hemorrhagic Fever (CCHF) is a severe and often fatal viral disease that poses a significant public health threat in various regions worldwide. Crimean-Congo hemorrhagic fever virus (CCHFV) is responsible for the disease, which can be passed to humans by tick bites or direct contact with infected animal blood and tissues. Eid-al-Adha, a highly esteemed religious festival in Iraq, encompasses the ceremonial sacrifice of livestock, potentially leading to favourable conditions for the transmission of CCHF. Here, the probable increase in CCHF incidence in Iraq after Eid-al-Adha in 2023 and the need to take preventative actions to halt the outbreak are discussed.

Muslims worldwide celebrate Eid al-Adha, also known as the “Sacrifice Feast,” to honour the Prophet Ibrahim's (Abraham) willingness to sacrifice his son. During this event, Iraqi Muslims slaughter sheep, goats, and cows as part of Qurbani (sacrifice). As these animals may contain ticks carrying CCHF, killing, and handling them might spread the illness. Human exposure to the virus may also arise via contact with infected animals' blood or tissues, especially if proper precautions are not taken.

Historical evidence and epidemiological research have linked Eid-al-Adha celebrations to CCHF in Muslim-populated regions [1]. Several neighbouring countries, including Iran and Turkey, have reported significant outbreaks after the festival [2]. Based on the most recent World Health Organization (WHO) Report dated August 14th, 2022 [3], there were 53 deaths out of the 295 laboratory-confirmed cases (Fig. 1). Since the beginning of 2023, the Iraqi Ministry of Health has reported over 250 verified cases of CCHF and 35 deaths [4]. The rise in CCHF cases over this period emphasizes the need for attention and prevention.

**Fig. 1:**
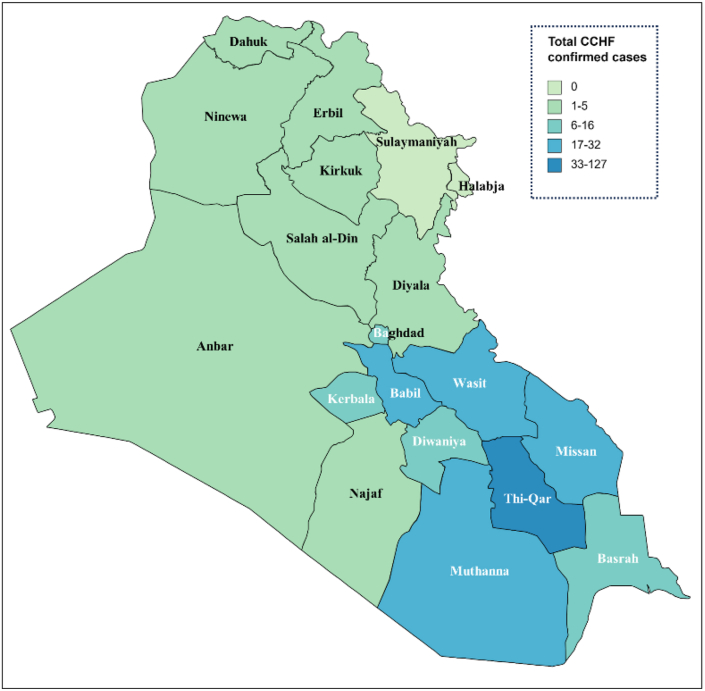
The distribution of Laboratory-confirmed cases of CCHF across the Iraqi governorates.

Multiple factors contribute to the heightened occurrence of CCHF following the celebration of Eid-al-Adha in Iraq. The significant quantity of animals sacrificed during the Eid-al-Adha religious festival fosters an environment conducive to the transmission of CCHF. Tick bites and exposure to contaminated blood and tissues may increase with animal handling and transportation. Despite awareness campaigns, some communities may not comprehend CCHF or take necessary preventive steps. Due to this lack of understanding, possible cases may be reported late, delaying effective control actions. Insufficient adherence to hygienic practices, such as inadequate sanitation protocols during animal slaughter, improper disposal methods for the animal remains, and the absence of adequate personal protective equipment (PPE), can also increase the likelihood of CCHF transmission to individuals involved in the sacrificial process.

To address the potential rise in CCHF cases during the Eid-al-Adha festival, it is imperative to implement a range of protective measures. Implementing extensive awareness campaigns is crucial to educate communities about CCHF, its modes of transmission, and preventive measures. It is recommended that these campaigns prioritize the communication of the significance of maintaining proper hygiene practices, adopting preventive measures against tick bites, and adhering to PPE protocols when engaging in animal handling activities. Implementing training sessions for healthcare professionals, veterinarians, and individuals engaged in animal slaughter can serve as an additional preventive measure. Applying this strategy will result in the timely identification, notification, and proper handling of suspected cases of CCHF. In addition, it is imperative to enhance disease surveillance systems to efficiently detect and address suspected cases of CCHF [5]. Effective surveillance systems enable rapid containment and intervention. Targeted vector management in high-risk areas and animal populations requires cooperation between the appropriate authorities. Finally, CCHF endemics need a public health emergency response approach. The strategy should include risk assessment, resource allocation, case management, and dissemination of information.

The potential rise in the incidence of CCHF in Iraq after the celebration of Eid-al-Adha in 2023 is a matter of considerable apprehension within global public health. Immediate actions are necessary to increase public knowledge, execute preventative tactics, and strengthen monitoring efforts to reduce the likelihood of outbreaks. Public health authorities and stakeholders can efficiently protect communities and mitigate the transmission of this fatal disease by establishing partnerships and cooperation at various scales, including local, national, and international levels. The prioritization of research, resource allocation, and public health preparedness is of the utmost importance in addressing the potential challenges that may arise from CCHF following the observance of Eid-al-Adha festivities.

## Ethics approval and consent to participle

Not applicable.

## Consent for publication

Not applicable.

## Funding

The authors did not receive any financial support for this work.

## Authors’ contribution

Conception: SH and SKA, Supervision: SH, Manuscript preparation: SH and KQ; Manuscript editing: SH, SKA, and KQ, Manuscript review: All authors, Final approval of manuscript: All Authors.

## Declaration of competing interest

The authors declare no conflicts of interest.
